# BioUML—towards a universal research platform

**DOI:** 10.1093/nar/gkac286

**Published:** 2022-05-10

**Authors:** Fedor Kolpakov, Ilya Akberdin, Ilya Kiselev, Semyon Kolmykov, Yury Kondrakhin, Mikhail Kulyashov, Elena Kutumova, Sergey Pintus, Anna Ryabova, Ruslan Sharipov, Ivan Yevshin, Sergey Zhatchenko, Alexander Kel

**Affiliations:** Sirius University of Science and Technology, Sochi 354340, Russian Federation; Federal Research Center for Information and Computational Technologies, Novosibirsk 630090, Russian Federation; Budker Institute of Nuclear Physics SB RAS, Novosibirsk 630090, Russian Federation; Sirius University of Science and Technology, Sochi 354340, Russian Federation; Biosoft.ru, LLC, Novosibirsk 630058, Russian Federation; Novosibirsk State University, Novosibirsk 630090, Russian Federation; Sirius University of Science and Technology, Sochi 354340, Russian Federation; Federal Research Center for Information and Computational Technologies, Novosibirsk 630090, Russian Federation; Budker Institute of Nuclear Physics SB RAS, Novosibirsk 630090, Russian Federation; Sirius University of Science and Technology, Sochi 354340, Russian Federation; Biosoft.ru, LLC, Novosibirsk 630058, Russian Federation; Federal Research Center for Information and Computational Technologies, Novosibirsk 630090, Russian Federation; Biosoft.ru, LLC, Novosibirsk 630058, Russian Federation; Biosoft.ru, LLC, Novosibirsk 630058, Russian Federation; Sirius University of Science and Technology, Sochi 354340, Russian Federation; Federal Research Center for Information and Computational Technologies, Novosibirsk 630090, Russian Federation; Sirius University of Science and Technology, Sochi 354340, Russian Federation; Sirius University of Science and Technology, Sochi 354340, Russian Federation; Sirius University of Science and Technology, Sochi 354340, Russian Federation; Biosoft.ru, LLC, Novosibirsk 630058, Russian Federation; Novosibirsk State University, Novosibirsk 630090, Russian Federation; Sirius University of Science and Technology, Sochi 354340, Russian Federation; Biosoft.ru, LLC, Novosibirsk 630058, Russian Federation; Sirius University of Science and Technology, Sochi 354340, Russian Federation; Biosoft.ru, LLC, Novosibirsk 630058, Russian Federation; Biosoft.ru, LLC, Novosibirsk 630058, Russian Federation; geneXplain GmbH, Wolfenbüttel 38302, Germany

## Abstract

BioUML (https://www.biouml.org)—is a web-based integrated platform for systems biology and data analysis. It supports visual modelling and construction of hierarchical biological models that allow us to construct the most complex modular models of blood pressure regulation, skeletal muscle metabolism, COVID-19 epidemiology. BioUML has been integrated with git repositories where users can store their models and other data. We have also expanded the capabilities of BioUML for data analysis and visualization of biomedical data: (i) any programs and Jupyter kernels can be plugged into the BioUML platform using Docker technology; (ii) BioUML is integrated with the Galaxy and Galaxy Tool Shed; (iii) BioUML provides two-way integration with R and Python (Jupyter notebooks): scripts can be executed on the BioUML web pages, and BioUML functions can be called from scripts; (iv) using plug-in architecture, specialized viewers and editors can be added. For example, powerful genome browsers as well as viewers for molecular 3D structure are integrated in this way; (v) BioUML supports data analyses using workflows (own format, Galaxy, CWL, BPMN, nextFlow). Using these capabilities, we have initiated a new branch of the BioUML development—u-science—a universal scientific platform that can be configured for specific research requirements.

## Graphical Abstract

**Graphical Abstract:**
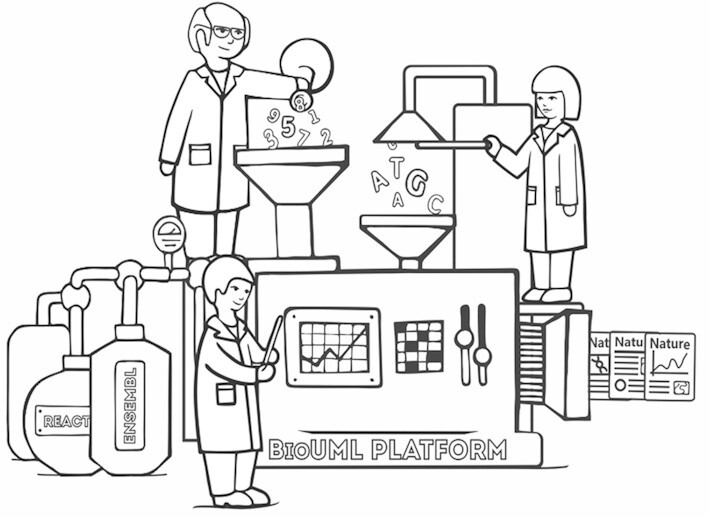
BioUML is a complex web-based platform for systems biology and data analysis integrated with Jupyter notebook, R/Bioconductor, Galaxy and Docker technologies.

## INTRODUCTION

BioUML (Biological Universal Modelling Language; https://www.biouml.org/)—is a web-based integrated platform for systems biology and data analysis. The major changes since the previous publication (1) are related with using new tools and technologies:

- Docker (https://www.docker.com/, (2))—is a software framework for building, running and managing containers on servers and the cloud. The major change in the BioUML architecture is related to usage of docker images whenever possible. It allows us to adopt the BioUML platform to state-of-the-art server infrastructure (like kubernetes), simplify its deployment as well as provide BioUML extensibility by new tool for data analyses. We have also developed some specification for metadata about docker image to describe programs and Jupyter kernels provided by this image (http://wiki.biouml.org/index.php/Docker_meta).
- Jupyter notebook/lab/hub (https://jupyter.org/, (3))—Jupyter notebooks are widely used in biosciences for interactive and reproducible data analysis and visualization. We have integrated Jypyter notebooks into the BioUML platform using the Jupyter hub technology and developed Jupyter kernels for R, Python and JavaScript languages that provide access to BioUML functionality inside corresponding notebooks.
- Git (https://git-scm.com, (4))—is a free and open source distributed version of the control system. Currently, the BioUML platform allows using Git repositories as projects where users can store their models, data, Jupyter notebooks, etc.
- Binder (https://mybinder.org/, (5))—is a technology that enables building of docker images from Git repositories and running Jupyter notebooks located there. We have also integrated BioUML with Binder to enable one to run built models anywhere either in the BioUML platform or as a Jupyter notebook with a single click.

Next changes are related to the usage and support of other tools and formats for data analyses using workflows:

- CWL—Common Workflow Language (https://www.commonwl.org/, (6))—is a YAML-based format to describe command line tools and connect them together to create workflows. The BioUML platform provides the web interface to specify CWL analysis parameters, visualize CWL workflows as diagrams and start them on the server using CWLRunner (http://wiki.biouml.org/index.php/CWL).
- Nextflow (https://www.nextflow.io/, (7))—is a domain specific language and execution environment for scalable and reproducible scientific workflows using software containers. The BioUML platform may use Nextflow to launch programs for data analyses on clouds and clusters.
- Galaxy (https://galaxyproject.org/, (8))—is an open-source platform for FAIR data analysis using workflows. Updated version of the BioUML platform is integrated with the Galaxy server using its REST API. Galaxy analyses can be displayed in the BioUML web interfaces, used in the BioUML workflows and executed using Galaxy (http://wiki.biouml.org/index.php/Galaxy).
- Galaxy Tool Shed (https://toolshed.g2.bx.psu.edu/, (9))—serves as an ‘appstore’ for the Galaxy platform and is a free service for tool developers and Galaxy administrators to host and share Galaxy utilities. The BioUML administrator web interface includes possibilities to configure a set of tools used by the installed Galaxy platform (http://wiki.biouml.org/index.php/Galaxy_admin).
- BPMN - Business Process Model and Notation (https://www.omg.org/spec/BPMN/, (10))—is a graphical representation for specifying business processes in a business process model. It is also widely used for microservices orchestration. We are experimenting to use this approach for development of workflows for NGS data analyses (http://wiki.biouml.org/index.php/BPMN). In comparison to common formats (CWL, Nextflow), it provides new workflow elements: logical gateways, events, compensatory action in case of any error. Moreover, BPMN allows to integrate program calls and user actions in one workflow (e.g., a user can enter additional parameters during the workflow execution). We use Camunda (https://camunda.com/, (11)) as BPMN engine and bpmn-js (https://bpmn.io/toolkit/bpmn-js/) as the JS library for BPMN diagram editing in the BioUML web interface.

We also significantly improved the BioUML genome browser. Currently it supports:

- BigBed and BigWig formats. Data in these formats can be displayed both in a genome browser and in tabular form;
- remote usage of files in the BigBed and BigWig formats listed above using http;
- track finder—is a new tab for finding needed tracks in the specified database. It is especially useful for databases like GTRD, which contains 150 000+ tracks;
- combined tracks—is a special type of track that can logically combine information from several tracks (http://wiki.biouml.org/index.php/Logical_track). For example, such a track can visualize sites that are common (overlaps) for a set of specified tracks. It should be noted that required calculations occur on the fly for the selected region in the genome browser;
- url references—user can specify region and track that should be visualized in the genome browser. To fine tune visualized tracks the genome browser accepts a JSON file (or string) (http://wiki.biouml.org/index.php/Genome_browser_json);
- integration to other web-pages—using a special iframe the BioUML genome browser can be integrated into other web pages (http://wiki.biouml.org/index.php/Genome_browser_iframe) and JSON described above can be used for its fine-tuning.

We have also integrated a viewer for molecular 3D structure (http://wiki.biouml.org/index.php/3D_viewer).

There are also a number of minor improvements and bug fixes (http://wiki.biouml.org/index.php/BioUML_development_history) as well as a number of new analyses related to the analysis of NGS data and transcription regulation.

On the base of the BioUML platform, several other bioinformatics platforms are developed:

- geneXplain (https://genexplain.com/genexplain-platform/)—is an online toolbox and workflow management system for a broad range of bioinformatics and systems biology applications. The main advantages of the geneXplain platform are:
- many sophisticated workflows for omics data analysis;
- fully integrated upstream analysis that combines state-of-the-art analysis of regulatory genome regions with sophisticated pathway analyses;
- integration with many databases for pathway analysis both public (Reactome (11), HumanCyc (12), GeneWays (13) and others) and commercial (TRANSFAC^®^ (14) and TRANSPATH^®^ (15));
- Genome Enhancer (https://genexplain.com/genome-enhancer/)—a fully automated pipeline for patient omics data analysis, which identifies prospective drug targets and corresponding treatments by reconstructing the molecular mechanism of the studied pathology. Proven applications of Genome Enhancer include cancer, neurodegenerative diseases, infectious diseases, diabetes and metabolic diseases;
- Sirius (https://sirius-web.org/)—an information platform for projects related to data analysis and modelling for educational and research projects of the Sirius University. This platform combines the possibilities of the BioUML platform for modelling and data analysis and GitLab for project storage, management and documentation;
- c-tau (https://ctd.inp.nsk.su/c-tau/)—a specialized platform for modelling and data analysis in high energy physics which was created on the base of the BioUML platform in collaboration with the Budker Institute of Nuclear Physics of the Siberian Branch of the Russian Academy of Sciences.

The last example demonstrates how the BioUML platform core can be used and extended for other problem domains. Thus, we have initiated a new branch of the BioUML development—u-science—a universal research platform that can be configured for specific research requirements.

## MATERIALS AND METHODS

### Architecture overview

**Figure 1.**
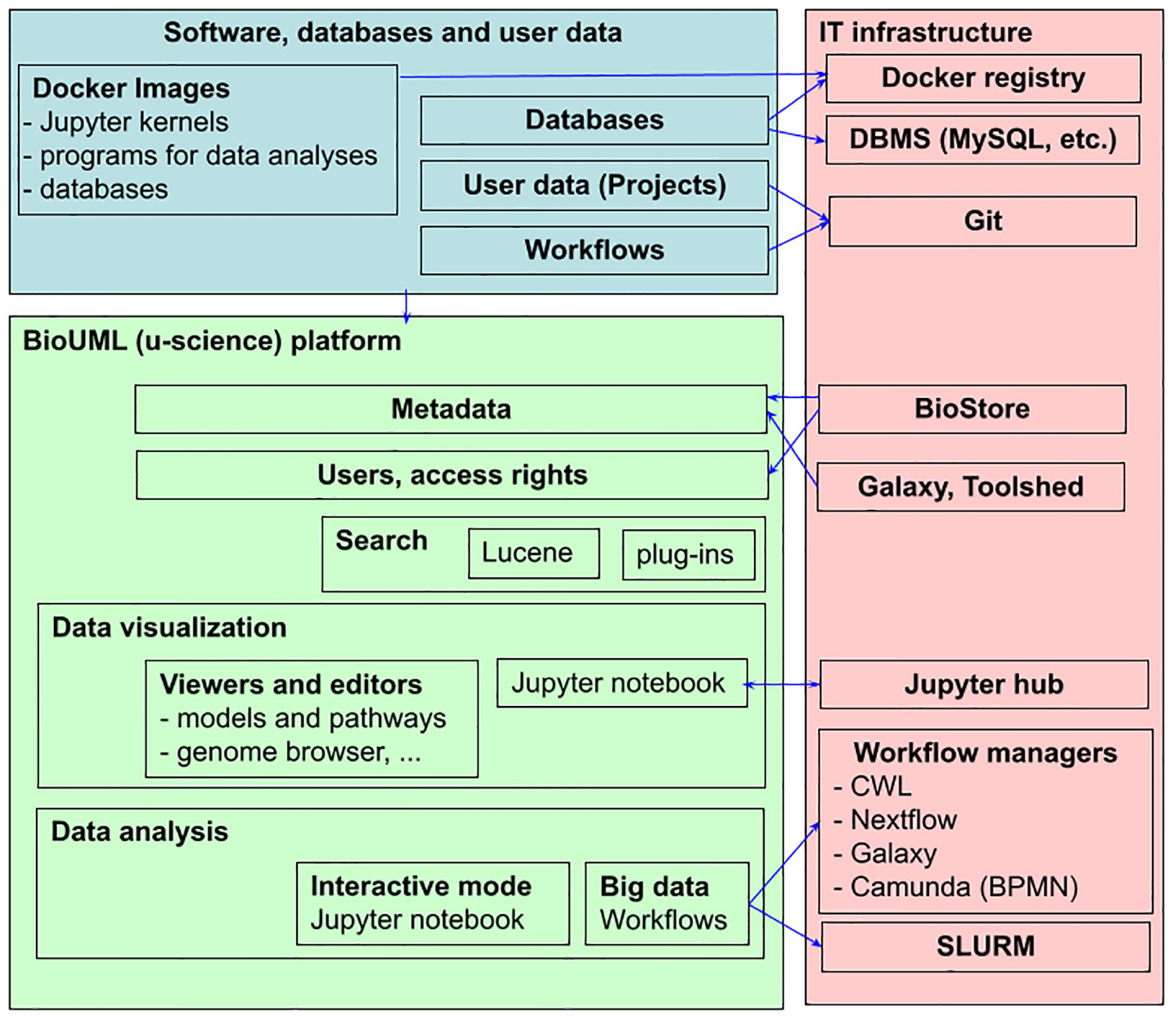
Architecture of the BioUML (u-science) platform. See its detailed description in the main text below.

Figure 1 demonstrates the updated structure of the BioUML platform and its integration and interactions with other tools and technologies.

Docker images—all software is containerized into docker images. These images are stored in docker registries. Both public (like https://hub.docker.com/) and private docker registries can be used. Databases together with their database management systems (DBMS) also can be containerized. We are using this approach on some BioUML server installations for the Ensembl databases. A number of docker images for specified organisms and Ensembl database versions together with MySQL DBMS were created.

User data is organized into projects. Such projects can correspond to Git repositories, so users can use some Git tools (for example, GitHub or GitLab) for project data management and documentation and the BioUML platform for modelling and data analysis. Test cases below will demonstrate this approach. Integration of GitLab and BioUML is also a core of the Sirius platform.

Git is often used to store workflows (for example, https://view.commonwl.org/workflows). It is especially useful while in this case Git repository usually contains not only the workflow code but also the workflow documentation and examples of input and output data.

BioStore (https://bio-store.org/) is the central hub which provides collaborative research using the BioUML platform. For this purpose, it contains a registry of users, their projects, access rights to projects (several users can have access to the same project) and list of publicly available servers where the BioUML platform or its derivatives (geneXplain, Genome Enhancer, Sirius) are installed (currently 15+ installations).

Special search engine based on Apache Lucene (https://lucene.apache.org/) is used to search information in the installed databases as well as to search needed analysis methods on the base of their meta-information (name, description, etc.).

The BioUML platform has a plug-in architecture based on Equinox OSGi (https://www.eclipse.org/equinox/). It allows you to specify extension points and different plug-ins can contribute to them. By this way, a different menu actions, panels, viewers and editors can be added to the platform. List of available extension points: http://wiki.biouml.org/index.php/Category:Extension_points.

BioUML provides a number of viewers and editors for data visualization:

- the diagram editor for visual construction of complex biological models using established and well-known standards: SBML (Systems Biology Markup Language) and SBGN (Systems Biology Graphic Notation). We have developed a special extension for SBGN for visual presentation of mathematical elements (http://wiki.biouml.org/index.php/SBGN_extension);
- pathway editor allows to visualize biological pathways from different databases (Reactome, TRANSPATH, etc.) using SBGN. SBGN-ML, SBML and BioPAX are supported. For KEGG pathways own graphic notation is used - it mimics original KEGG pathway visualization;
- powerful genome browser, its improvements were described above;
- viewer for molecular 3D structures;
- data viewer and editor for tabular data;
- code viewers and editors (JavaScript, R);
- standard viewers for HTML documents;
- Jupyter notebooks (see below).

Data analysis can be done interactively using Jupyter notebooks or using workflows. In the latter case, different workflow executors can be started on clouds and clusters (see Introduction).

### Jupyter notebook/lab/hub

Jupyter is a widely used tool for interactive reproducible analysis and data visualization. It supports multiple programming languages. The kernel concept implying a launch of a specified kernel on the server to perform the analysis. The web-interface (a notebook) interacts with the kernel using a special API and transfers data using JSON format.

The Jupyter hub technology enables to set a number of kernels for interactive commands execution and results representation in the web-interface. Each kernel is represented by a docker image and described in Jupyter hub settings.

BioUML provides docker image with several BioUML-aware kernels along with all popular scientific libraries used in the specific language.

Currently, BioUML provides the following Jupyter kernels:

- Python 3—the kernel for Python 3 programming language. It also includes pybiouml library (https://github.com/Biosoft-ru/pybiouml) that provides access from Python scripts to user data, analysis methods and workflows on BioUML servers.
- R (BioUML)—the kernel for R programming language. It also includes rbiouml library (https://cran.r-project.org/package=rbiouml) that provides access from R scripts to user data, analysis methods and workflows on BioUML servers.
- JS (BioUML)—the kernel for JavaScript programming language. It uses the Rhino library from Mozilla (https://github.com/mozilla/rhino) to execute JavaScripts in the Java environment. Special instance of the BioUML server is used as the kernel, so this kernel provides seamless integration with all functions of BioUML. From one hand, BioUML provides JavaScript API (JavaScript functions and host objects) for main functions related to the modelling and data analysis. On the other hand, the Rhino library provides access to all Java code inside BioUML. However, there are some restrictions related to security reasons. For example, direct access to the file system is prohibited. Special Java security manager configured and used for this purpose.
- SoS - Script of Scripts—is a special kernel (https://vatlab.github.io/sos-docs/, (16)) that allows execution of scripts in multiple languages in a single Jupyter notebook, with seamless integration of multiple Jupyter kernels (e.g., python and R).

New Jupyter kernels can be added. For this purpose, the server administrator should put the corresponding Docker image to the Docker registry that is connected with the BioUML server.

## USE CASES

### Modular modelling of complex biological systems

Biological models have been becoming and are supposed to be more complex as well as multiscale. The most efficient strategy to deal with the complexity that is known from ancient times—divide and conquer. In the case of biological models, the modular modelling is a suggested approach to overcome the complexity and hierarchical structure of biological systems. SBML comp package is the great effort in this direction that provides the required standard (17). We also need a common repository for modules that can be reused in many complex models (18).

Nevertheless, composite models are still rarely used in systems biology now. This is one of the main reasons why biological models are so small and simple in comparison with engineering models and software programs. So, we need to employ practices from these fields.

To achieve the goal, we combine three technologies in the Sirius platform: (i) GitLab that is widely used in software development for project storage, management and documentation, (ii) BioUML for visual modular modelling and (iii) Jupyter notebooks for result visualization and reproducible research. It allows us to construct complex modular models of blood pressure regulation, skeletal muscle metabolism and COVID-19 epidemiology. Their brief description, summary statistics and references to corresponding Git repositories and original articles are shown below.

### Multiscale model of dynamic changes in human skeletal muscle during the physical exercise and recovery

https://gitlab.sirius-web.org/virtual-patient/muscle-metabolism

25 modules, 238 species,185 reactions, 171 ordinary differential equations, 647 parameters.

To systematically investigate the signalling-metabolic pathways relationships with downstream genetic regulation in the skeletal muscle, we built, for the first time, the modular model that describes exercise-induced changes in metabolic, signalling and gene expression levels and consists of 25 mathematical modules including blood compartment, muscle cell compartment with corresponding subcellular modules, transport modules for oxygen delivery and metabolites transport between muscle cells and capillary blood as well as between intracellular compartments and taking into account two types of the fibres that comprise the human skeletal muscle tissue (19). The integrated modular model validated on diverse including original experimental data and different exercise modes provides a comprehensive *in silico* platform in order to decipher and track cause–effect relationships between metabolic, signaling and gene expression levels in skeletal muscle.

### Agent-based modular model of blood pressure regulation

https://gitlab.sirius-web.org/virtual-patient/blood-pressure-regulation

20 modules, 25 ordinary differential equations, 160 algebraic expressions, 132 parameters, 160 variables, 10 discrete events.

This is a modular agent-based mathematical model of the human cardiovascular and renal systems (20). We performed the model calibration to find an equilibrium state within the normal vital sign ranges for a healthy adult. We verified the model's abilities to reproduce equilibrium states with abnormal physiological values related to different combinations of cardiovascular diseases (such as systemic hypertension, chronic heart failure, pulmonary hypertension, etc.). For the model creation and validation, we involved over 200 scientific studies covering known models of the human cardiovascular and renal functions, biosimulation platforms, and clinical measurements of physiological quantities in normal and pathological conditions. We compiled detailed documentation describing all equations, parameters and variables of the model with justification of all formulas and values.

### COVID-19 epidemiology and immunology

https://gitlab.sirius-web.org/covid-19/dde-epidemiology-model

https://ict.biouml.org/bioumlweb/#de=data/Collaboration/Covid-19/

A series of mathematical models describing COVID-19 epidemiology and immunology was developed using the BioUML platform. We have used an iterative approach for models development similar to agile methodologies used in software development (https://www.agilealliance.org/agile101/). We have started from the model suggested by Westerhoff and Kolodkin (21). During the second iteration we have added the Stringency Index (22) as a representation of government non-pharmaceutical measures and vaccination. The third version of the model (23) employs delay differential equations to describe transitions between different stages like incubation period, symptoms onset, recovery, etc. Each transition is described by a weighted sum of arguments with different time delays. Weights are estimated to fit real processes better than it can be done with mass-action laws usually used in SEIR models. The fourth version takes into account loss of immunity after recovery. All models were developed as modular diagrams containing one submodel which represents the core model with all transitions and states and top level model to define the context (a particular region, government measures, import from other regions, testing policy and vaccination). In that way the one core model can be placed in different contexts and vice versa different core models (with different formalisms and detailed levels) can be placed in the same context.

A web tool based on the BioUML and developed models provides users with an interface for simulating different scenarios of COVID-19 epidemic in chosen regions: https://covid19.biouml.org/.

### Flux balance analysis

BioUML also provides an opportunity to conduct the analysis in order to simulate the flux distribution for genome-scale metabolic models. The guideline and some examples of the model analysis are presented in our wiki: http://wiki.biouml.org/index.php/Flux_balance_analysis. In short, there are three alternative ways to launch the flux balance analysis in BioUML:

- via special Flux Balance tab in the Operations field of the diagram;
- via corresponding Flux Balance Constraint method presented in the Analysis tab of the Tree area (see Get started in the guideline).
- using the Jupyter notebook that includes the well-known COBRAPy package (24). As an example, the Jupyter notebook has been created to run the *iMK1321* metabolic model (see wiki page above; (25)) using GLPK (GNU Linear Programming Kit) solver (http://www.gnu.org/software/glpk).

### Analysis of NGS data

The BioUML platform is used for uniform analyses of the next generation sequencing (NGS) data related with gene transcription regulation for the GTRD database (26). For this purpose, a number of specialized analyses methods and workflows were developed. We also have developed a special perspective in the BioUML platform (http://gtrd.biouml.org) that provides browsing and displaying information, advanced search possibilities, integration of the genome browser to visualize the GTRD data and information from the Ensembl database.

The geneXplain platform, which was built on the basis of BioUML, is used to analyse various NGS with the help of specific ‘upstream analysis’ pipeline (27). This approach combines statistical analysis of NGS data, computing differentially expressed genes, analysis of their promoters and identification of key master regulators in the gene regulatory network. With the help of this approach, NGS data for various diseases were successfully analyzed: resistant to methotrexate in colon cancer (28); pathways of ageing (29); for sensitivity to anticancer therapy in lung cancer (30).

### Analysis of multi-omics data

Recently a new software tool, Genome Enhancer was developed on the basis of the geneXplain platform and BioUML technology. It performs fully automated analysis of multi-omics NGS data that may include any combinations of five omics types: transcriptomics (RNA-seq, microarrays), epigenomics (ChiP-seq, ATAC-seq, DNA methylation assays), proteomics (shotgun ms-based, phosphoproteomics), genomics (NGS full genome, exome, GWAS) and metabolomics. It takes data in multiple formats and automatically generates hypotheses about molecular mechanisms of studied pathologies, finds most promising drug targets and biomarkers and suggests drugs and active compounds as treatment candidates for the pathology. With the help of Genome Enhancer, several successful studies were performed including: identification of early diagnostic biomarkers of colorectal cancer (31); study of potential mechanisms of atherosclerosis (32); drug repurposing in IBD (33).

### Platform extensions

BioUML plug-in architecture allows adding new viewers and editors. For example, new viewer and new editor were added in the updated version:

- viewer for molecular 3D structure: http://wiki.biouml.org/index.php/3D_viewer
- BPMN editor: http://wiki.biouml.org/index.php/BPMN

### Other problem domains

Based on the BioUML platform, we have developed a specialized platform for the Super charm-tau factory, the large scale high energy physics collider experiment. The platform allows for user-friendly simulation, analysis and visualization of data using experiment-specific software. The platform includes a biomedicine non-specific functionality of the BioUML platform. In addition, it includes the next features:

- Root (http://root.cern.ch)—a set of tools to work with large amounts of data.
- Special type of the diagram for visualization and simulation of imitation models of c-tau factory. The model describes computations and data storage necessary for c-tau factory functioning.
- Common Workflow Language (CWL, www.commonwl.org)—a standard for the description and execution of arbitrary command line tools and workflows. The platform provides convenient user interface for CWL.
- Ability to run analyses, CWL-tools and workflows in the docker as well as remote servers using SLURM manager (https://github/SchedMD/slurm).

The platform is described in details at http://wiki.biouml.org/index.php/c-tau.

## DISCUSSION

Comprehensive feature comparison of the BioUML platform with other widely used tools for systems biology (CellDesigner (34), Tellurium (35), COPASI (36), iBioSim (37) and other), pathway visualization and analysis (Cytoscape (38), etc.) are available at http://wiki.biouml.org/index.php/Tools_Comparison_2022.

Despite the significant difference in data, programs and methods for their analysis and visualization in different problem domains it is possible to create a universal research platform that will provide:

- following the principles of FAIR—Findability, Accessibility, Interoperability and Reusability (39);
- reproducible research (40);
- collaborative research;
- convenient web interface for data search, analysis and visualization.

Such universal research platform is based on three principles:

- We are not going to replace or recreate the well-established tools that have already been built in each problem domain, but we would like to put them in a single context where they can be easily found and used.
- To formalize, uniformly describe and structure the data, programs and methods existing in the problem domain for their analysis and visualization.
- There are a number of routine IT tasks that are not specific to the problem domain, but require a lot of efforts to solve them:
- management of access rights to data and programs;
- access control to IT resources, task queue management;
- enabling collaboration on data analysis;
- web interface for access to data and programs;
- version control system for data and programs.

These tasks can be effectively resolved within the framework of a universal research platform. A number of BioUML-derived platforms (geneXplain, Genome Enhancer, Sirius, c-tau) shows that it already contains all required components. However, it requires some additional work to make the BioUML core more suitable for further extensions and configurations. So, we have initiated a new branch of the BioUML development—u-science—a universal research platform that can be configured for specific research requirements.

## DATA AVAILABILITY

The BioUML freely accessible web server is available at https://biouml.org.

Additional documentation is available at https://wiki.biouml.org.

The Sirius platform: https://sirius-web.org.

The BioUML web interface is compatible with main commonly-used web browsers: Chrome, Firefox, and Microsoft Edge.
